# Vertical Mandibular Range of Motion in Anesthetized Dogs and Cats

**DOI:** 10.3389/fvets.2016.00051

**Published:** 2016-06-28

**Authors:** Margherita Gracis, Eric Zini

**Affiliations:** ^1^Istituto Veterinario di Novara, Granozzo con Monticello, Novara, Italy; ^2^Clinica Veterinaria San Siro, Milano, Italy; ^3^Vetsuisse Faculty, Clinic for Small Animal Internal Medicine, University of Zurich, Zurich, Switzerland; ^4^Department of Animal Medicine, Production and Health, University of Padova, Legnaro, Padova, Italy

**Keywords:** range of motion, mandible, temporomandibular joint, dogs, cats

## Abstract

The main movement of the temporomandibular joint of dogs and cats is in vertical dimensions (opening and closing the mouth). An objective evaluation of the vertical mandibular range of motion (vmROM) may favor early diagnosis of a number of conditions affecting the joint mobility. vmROM, corresponding to the maximum interincisal opening, was measured in 260 dogs and 127 cats anesthetized between June 2011 and April 2015 because of oral or maxillofacial problems and procedures. Animals with a known history of or having current diseases considered to hamper mandibular extension were excluded from the study. Dogs were divided into four subgroups, based on body weight: subgroup 1 (≤5.0 kg, 51 dogs), subgroup 2 (5.1–10.0 kg, 56 dogs), subgroup 3 (10.1–25 kg, 66 dogs), and subgroup 4 (>25.1 kg, 87 dogs). The mean vmROM of all dogs was 107 ± 30 mm (median 109, range 40–180); in subgroup 1 was 67 ± 15 mm (median 67, range 40–100), in subgroup 2 was 93 ± 15 mm (median 93, range 53–128), in subgroup 3 was 115 ± 19 mm (median 116, range 59–154), and in subgroup 4 was 134 ± 19 mm (median 135, range 93–180). The mean vmROM of the cats was 62 ± 8 mm (median 63, range 41–84). Correlations between vmROM, age, sex, and body weight were evaluated. In dogs, vmROM did not correlate with age, and in cats a weak positive correlation was found. vmROM and body weight were positively correlated in both populations, except dog subgroup 2. Overall, mean vmROM and body weight were significantly higher in male than in female, both in dogs and in cats. However, vmROM did not differ between sexes in any of the canine subgroups, and only in subgroup 4 male dogs were significantly heavier than females. Evaluation of vmROM should be incorporated into every diagnostic examination as it may be valuable in showing changes over time for every single patient.

## Introduction

The temporomandibular joint (TMJ) is a synovial condylarthrosis composed by the mandibular head of the condylar process and the mandibular fossa of the squamous part of the temporal bone. Joint mobility is favored by the action of the masticatory muscles, including the masseter, the temporal, the medial and lateral pterygoid, and the digastricus muscles. They all originate from the skull, attach to the caudoventral region of the mandible and, except the digastricus muscle, act adducting or raising the mandible, therefore closing the mouth. The digastricus muscle is responsible for opening the mouth, together with the force of gravity. Mild diduction (small lateral movements) is favored by contraction of the lateral pterygoid and deep part of the masseter muscles (1–3). However, because of the shape and angulation of the condylar process of the mandible, in dogs and cats, the main mandibular movement is in vertical dimensions (opening and closing the mouth), with little lateral range of motion (ROM) (3, 4).

Mandibular ROM may be hampered or limited by a number of conditions affecting the intrarticular or extrarticular structures, such as true or false TMJ ankylosis, ostheoarthirtis, fracture, osteomyelitis, bone neoplasia, retrobulbar masses, neuromuscular diseases and trismus (e.g., masticatory myositis, tetanus), craniomandibular osteopathy, and others (5–12). Early detection of reduced ROM may therefore allow for early diagnosis of these diseases. However, the normal ROM of the TMJ in dogs and cats is currently unknown.

The main aim of this prospective study was to assess the mandibular ROM in vertical dimension (i.e., opening of the mouth) in anesthetized dogs and cats. Furthermore, the influence of age, sex, and body weight on mandibular ROM was investigated. It was hypothesized that immature animals had a relatively higher degree of motion than older animals.

## Materials and Methods

### Case Inclusion

All animals included in this study were client-owned dogs and cats anesthetized between June 2011 and April 2015 because of oral or maxillofacial problems and procedures. Animals with a known history of or having current diseases considered to hamper mandibular extension (e.g., true or false ankylosis, intrarticular or periarticular neoplasia, maxillofacial trauma) were excluded from the study. Data collected for each animal included signalment (age, sex, breed, and body weight) and measurements of vertical mandibular range of motion (vmROM). Dogs were divided into four subgroups, based on body weight: subgroup 1 (≤5.0 kg), subgroup 2 (5.1–10.0 kg), subgroup 3 (10.1–25 kg), and subgroup 4 (>25.1 kg).

### Vertical Mandibular Range of Motion Measurement

**Figure 1:**
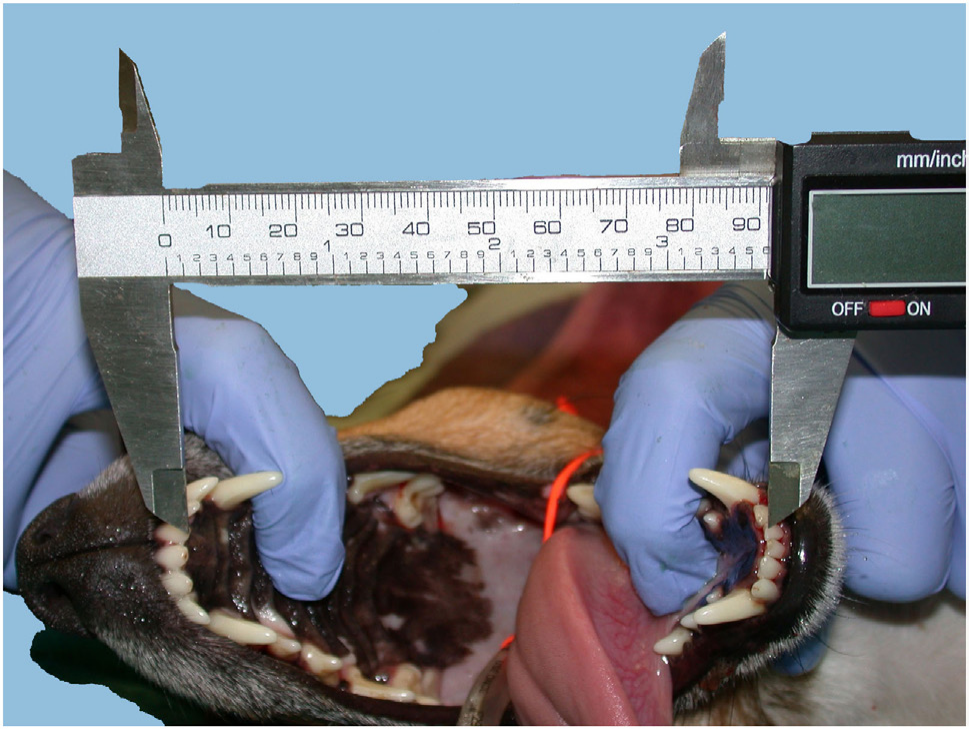
**vmROM measurement between the left maxillary and mandibular first incisor teeth in a dog, using a caliper**.

Vertical mandibular range of motion corresponded to the maximum interincisal opening and was measured with an endodontic ruler or a precision caliper between the incisal edge of the mandibular and maxillary incisor teeth, while a helper maximally extended the mandibles, firmly holding the mandibular and maxillary bones (i.e., passive vmROM) (Figure 1). The measurement was rounded to the closest millimeter. vmROM was measured between the most mesial corresponding mandibular and maxillary incisor tooth present on either right or left side (i.e., right or left mandibular and maxillary first incisor teeth or, if any of these were missing, right or left mandibular and maxillary second incisor teeth, or if any of these were also missing, right or left mandibular and maxillary third incisor teeth). If none of the corresponding incisor teeth were present, the measurement was taken at the gingival margin.

Although the timing was not standardized, vmROM was measured within 20 minutes from induction and endotracheal intubation, after a deep plane of anesthesia was reached and following systematic clinical evaluation of extraoral and intraoral structures.

### Statistical Analysis

Data are reported as mean ± SD as well as median and range. The Kolmogorov–Smirnov test was used to assess normality of all data sets. In dogs, the four subgroups divided according to body weight were studied to verify whether age and sex distribution were similar; age was compared with one-way ANOVA followed by Bonferroni *post hoc* test, and sex (female, male) distribution was compared with *r* × *c* contingency tables. Within each subgroup, associations between vmROM and age or body weight were investigated with Pearson’s correlation coefficient. Differences for vmROM between genders (female, male) were investigated with unpaired *t*-test. Body weight was compared between genders with unpaired *t*-test. In cats, associations between vmROM and age or body weight and differences for vmROM between genders (female, male) were investigated with the same tests. Body weight was compared between female and male cats with unpaired *t*-test.

Furthermore, for dogs and cats with vmROM measured more than once, at different times, comparisons between measurements were performed with paired *t*-test; if more than one measurement was available, it was arbitrarily chosen to retain the highest value in the analysis. A commercial software^1^ was used. Significance was set at *p* < 0.05; *p*-values were corrected with Bonferroni *post hoc* test due to repeated analyses.

## Results

### Dogs

Vertical mandibular range of motion was evaluated in 260 dogs, including 51 (19.6%) dogs in subgroup 1, 56 (21.5%) in subgroup 2, 66 (25.4%) in subgroup 3, and 87 (33.5%) in subgroup 4 (Table S1 in Supplementary Material). The number of dogs did not significantly differ between subgroups.

#### Age

**Figure 2:**
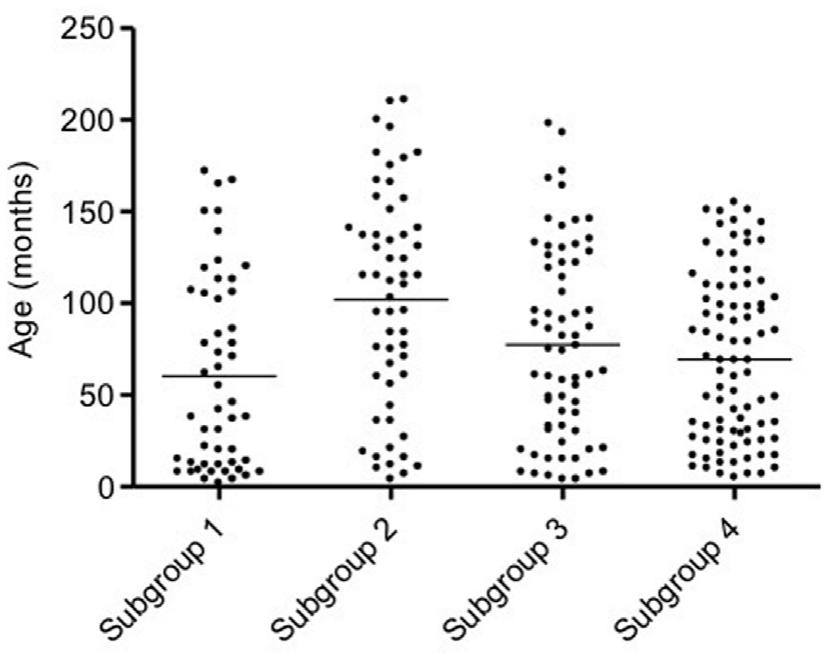
**Scatter dot plot of age in the four subgroups of dogs**. Dogs in subgroup 2 are significantly older than dogs in subgroups 1 and 4 (*p* < 0.001 and *p* < 0.01, respectively). The horizontal line depicts the mean.

Mean age of all dogs was 77 ± 54 months (median 75, range 2–211). In subgroup 1, the mean age was 60 ± 52 months (median 42, range 2–172), in subgroup 2 was 102 ± 59 months (median 111, range 4–211), in subgroup 3 was 77 ± 53 months (median 75, range 4–198), and in subgroup 4 was 69 ± 45 months (median 69, range 5–155). Dogs in subgroup 2 were significantly older than dogs in subgroup 1 (*p* < 0.001) and subgroup 4 (*p* < 0.01) (Figure 2), but not different as compared to those in subgroup 3. Age of dogs in subgroups 1, 3, and 4 did not differ.

Thirty-two dogs (12.3% of the entire canine population) were younger than 12 months of age: 13 dogs in subgroup 1, 5 in subgroup 2, and 7 each in subgroups 3 and 4. The number of young dogs did not differ significantly between the subgroups.

#### Sex

Overall, 119 (45.8%) dogs were female (42 intact and 77 neutered), and 141 (54.2%) were male (115 intact and 26 castrated). In subgroup 1, there were 23 (45.1%) females (14 intact and 9 neutered) and 28 (54.9%) males (24 intact and 4 castrated), in subgroup 2, there were 27 (48.2%) females (5 intact and 22 neutered) and 29 (51.8%) males (24 intact and 5 castrated), in subgroup 3, there were 36 (54.5%) females (8 intact and 28 neutered) and 30 (45.5%) males (26 intact and 4 castrated), and in subgroup 4, there were 33 (37.9%) females (15 intact and 18 neutered) and 54 (62.1%) males (41 intact and 13 castrated). Females and males were not differently represented among the four subgroups of dogs (*p* = 0.226).

#### Breeds

Overall, 53 (20.4%) were crossbreed dogs, and 107 (79.6%) purebreed dogs (Table 1).

**Table 1:** **Canine breeds of cases included in the study**.

Breeds	Number of animals (%)
Mixed breed	53 (20.4)
Labrador Retriever	18 (6.9)
Chihuahua	14 (5.4)
Dachshund	12 (4.6)
Boxer, Maltese	11 (4.2)
Golden Retriever	10 (3.8)
Cocker Spaniel, Miniature Poodle	9 (3.4)
Yorkshire Terrier	8 (3.1)
Jack Russell Terrier	7 (2.8)
Czechoslovakian Wolfdog	6 (2.3)
German Shepherd, Miniature Pinscher, Shi-Tzu	5 (1.9)
Border Collie, Rottweiler, English Setter	4 (1.5)
American Staffordshire, Australian Shepherd, Beagle, Bernese Mountain Dog, Bull Terrier, Dobermann Pinscher, Fox Terrier, Rhodesian Ridgeback	3 (1.1)
Alaskan Malamute, American Cocker, Argentine Dogo, Cavalier King Charles, French Bouledogue, Greyhound, Irish setter, Pitbull, Pugs, Spitz, West Highland Terrier	2 (0.8)
Akita Inu, Azawakh, Basset Blue de Guascogne, Belgian Malinoise, Belgian shepherd, Bobtail, English Bulldog, Epagneul Papillon, Flat-coated Retriever, Great Pyrenees Dog, Irish Wolfhound, Lagotto, Löwchen (Little Lion Dog), Maremma sheepdog, Pekingese Dog, Tibetan Terrier Dog, Toy Poodle, Weinmaraner, Zwergschnauzer	1 (0.4)

In subgroup 1, there were 2 (3.9%) crossbreed dogs and 49 (96.1%) purebreed dogs, including Chihuahua (14 cases); Maltese (10); Yorkshire terrier (6); Miniature Pinscher, Miniature Poodle (4); Jack Russell terrier, Shi-Tzu, Spitz (2); and Dachshund, Epagneul Papillon, Pekingese, Pugs, Toy Poodle (1).

In subgroup 2, there were 19 (33.9%) crossbreed dogs and 37 (66.1%) purebreed dogs, including Dachshund (10 cases); Jack Russell Terrier, Miniature Poodle (5); Shi-Tzu (3); Cavalier King Charles, West Highland terrier, Yorkshire terrier (2); and American Cocker, Fox terrier, French Bouledogue, English Bulldog, Löwchen, Maltese, Miniature Pinscher, Zwergshnauzer (1).

In subgroup 3, there were 24 (36.4%) crossbreed dogs and 42 (63.6%) purebreed dogs, including Cocker Spaniel (9 cases); Border Collie (4); Beagle, Bull Terrier, English Setter, Golden Retriever (3); Fox Terrier (2); and American Cocker, American Staffordshire, Australian Shepherd, Azawakh, Basset Blue de Guascogne, Belgian Shepherd, Dachshund, French Bouledogue, Irish Setter, Labrador Retriever, Lagotto, Pugs, Rhodesian Ridgeback, Rottweiler, Tibetan terrier (1).

In subgroup 4, there were 8 (9.2%) crossbreed dogs and 79 (90.8%) purebreed dogs, including Labrador retriever (17 cases); Boxer (11); Golden retriever (7); Czechoslovakian Wolfdog (6); German Shepherd (5); Bernese Mountain Dog, Dobermann Pinscher, Rottweiler (3); Alaskan Malamute, American Staffordshire, Argentine Dogo, Australian shepherd, Greyhound, Pitbull, Rhodesian ridgeback (2); Akita Inu, Belgian Malenoise; and Bobtail, English Setter, Flat-coated Retriever, Great Pyrenees Dog, Irish Setter, Irish Wolfhound, Maremma Sheepdog, Weinmaraner (1).

Crossbreed dogs were less represented in subgroup 1 (*p* < 0.001) and in subgroup 4 (*p* < 0.001).

#### Body Weight

Mean body weight of all dogs was 17.6 ± 13.1 kg (median 13.9, range 1.4–69.0). In subgroup 1, the mean body weight was 3.1 ± 1.0 kg (median 2.9, range 1.4–5.0), in subgroup 2 was 7.4 ± 1.5 kg (median 7.2, range 5.1–10.0), in subgroup 3 was 16.8 ± 5.0 kg (median 15.5, range 10.2–25.0), and in subgroup 4 was 33.4 ± 6.8 kg (median 32.0, range 25.5–69.0).

#### Vertical Mandibular Range of Motion

Mean vmROM of all dogs was 107 ± 30 mm (median 109, range 40–180).

In subgroup 1, the mean vmROM was 67 ± 15 mm (median 67, range 40–100), in subgroup 2 was 93 ± 15 mm (median 93, range 53–128), in subgroup 3 was 115 ± 19 mm (median 116, range 59–154), and in subgroup 4 was 134 ± 19 mm (median 135, range 93–180).

The minimum vmROM (40 mm) was recorded in a Chihuahua dog weighting 1.4 kg, one of the three lighter dogs of the studied population. The maximum vmROM (180 mm) was recorded in an Irish Wolfhound dog weighting 69 kg, the largest dog of the studied population.

Forty-five dogs had vmROM measured more than once (mean time interval between first and last visit: 7 months, median: 3 months, range: 1–38 months) (Table S2 in Supplementary Material). The mean vmROM difference between two examinations was 2 ± 8 mm (median 0, range −9 to 29). Seven of these dogs were younger than 1 year of age at the time of the first examination and were re-evaluated between 1 and 8 months later. By excluding these dogs from analysis, vmROM difference between two examinations was 1 ± 5 mm (median 0, range −9 to 11). For the young dogs, the mean difference in vmROM between the first and the last examination was 12 mm (median: 11 mm, range: −3 to 29 mm). Differences were not significant if the whole group was considered or in case young dogs were excluded (*p* = 0.100 and *p* = 0.475, respectively). Body weight did not differ between the two examinations, in either case. However, as expected, body weight significantly increased for the young dogs (*p* = 0.045).

Overall, no correlation was documented between vmROM and age (*p* = 0.956). Mean vmROM was significantly higher in male than female [male 112 ± 32 mm (median 115, range 43–180); female 102 ± 27 mm (median 101, range 40–161); *p* = 0.021] but did not differ between intact and castrated male (*p* = 0.490) or intact and spayed female (*p* = 0.631). Mean body weight was also higher in male than in female dogs (*p* < 0.013) [male 19.5 ± 14.4 mm (median 15.5, range 1.4–69.0); female 15.5 ± 10.9 mm (median 12.0, range 1.4–37.0)].

**Figure 3:**
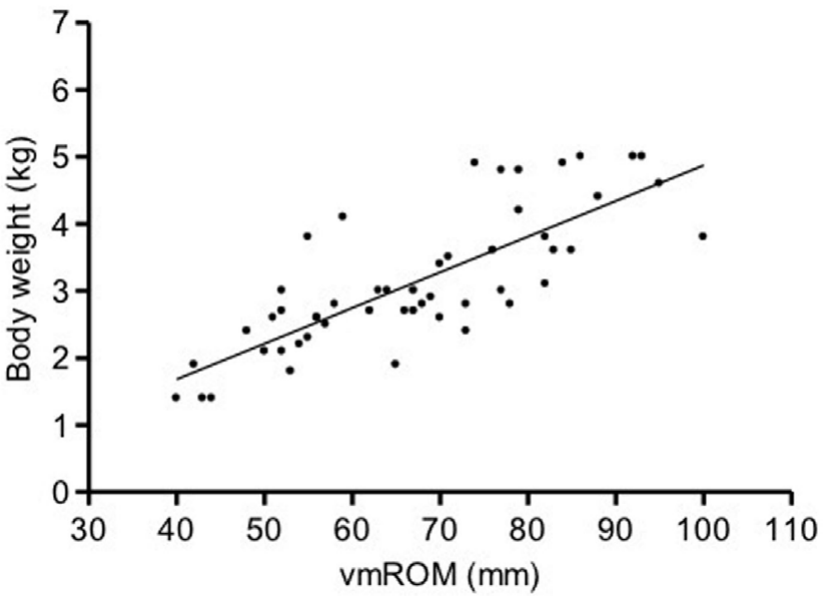
**Scatter dot plot of vmROM and body weight in dogs in subgroup 1**. A significant positive correlation is identified (*p* < 0.001). The regression line is shown.

In subgroup 1, a significant positive correlation was documented between vmROM and body weight (*r* = 0.80, CI 95%: 0.66–0.88, *p* < 0.001; Figure 3), but not between vmROM and age (*p* = 0.171). vmROM did not differ between male and female dogs (*p* = 0.243). Body weight did not differ between male and female dogs (*p* = 0.173).

**Figure 4:**
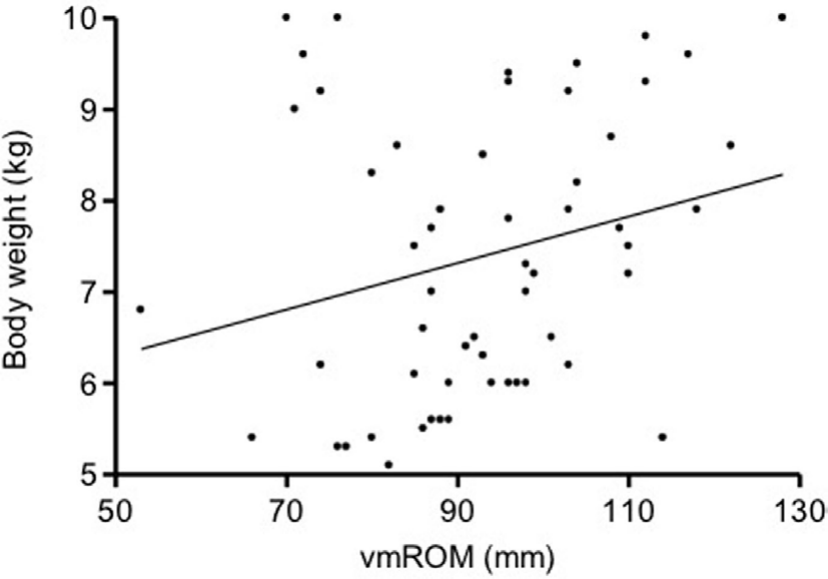
**Scatter dot plot of vmROM and body weight in dogs in subgroup 2**. A positive, but not significant, correlation is identified (*p* = 0.061). The regression line is shown.

In subgroup 2, correlations were not identified between vmROM and body weight (*p* = 0.061) (Figure 4) or between vmROM and age (*p* = 0.135). vmROM did not differ between female and male dogs (*p* = 0.701). Body weight did not differ between male and female dogs (*p* = 0.173).

**Figure 5:**
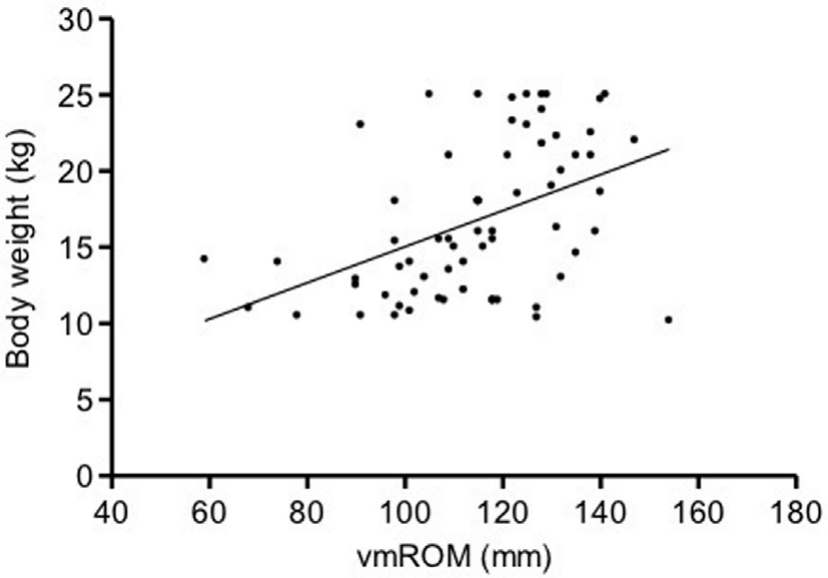
**Scatter dot plot of vmROM and body weight in dogs in subgroup 3**. A significant positive correlation is identified (*p* < 0.001). The regression line is shown.

In subgroup 3, a significant positive correlation was documented between vmROM and body weight (*r* = 0.45, CI 95%: 0.24–0.63, *p* < 0.001; Figure 5), but not between vmROM and age (*p* = 0.886). vmROM did not differ between female and male dogs (*p* = 0.545). Body weight did not differ between male and female dogs (*p* = 0.356).

**Figure 6:**
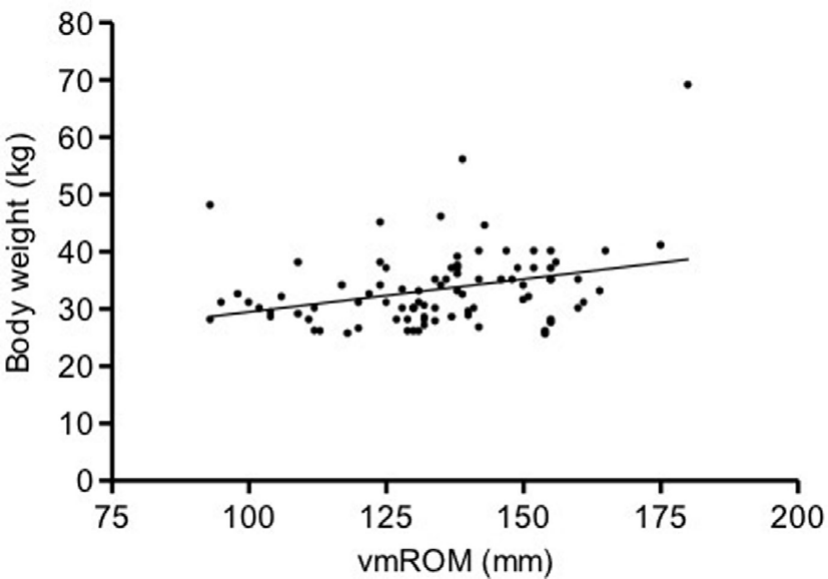
**Scatter dot plot of vmROM and body weight in dogs in subgroup 4**. A significant positive correlation is identified (*p* = 0.003). The regression line is shown.

In subgroup 4, a significant positive correlation was documented between vmROM and body weight (*r* = 0.32, CI 95%: 0.11–0.49, *p* = 0.003; Figure 6), but not between vmROM and age (*p* = 0.265). vmROM did not differ between female and male dogs (*p* = 0.545). Body weight was significantly heavier in male than female [male, 35.3 ± 7.7 kg (median 34.5, range 25.5–69.0); female 30.3 ± 3.3 kg (median 30.0, range 25.6–37.0); *p* = 0.003].

### Cats

Vertical mandibular range of motion was evaluated in 127 cats (Table S3 in Supplementary Material).

#### Age

Mean age of the cats was 75 ± 55 months (median 65, range 4–228). Twelve (9.4%) cats were younger than 12 months of age.

#### Sex

Seventy-six (59.8%) cats were male (6 intact and 70 castrated), and 51 (40.2%) were female (6 intact and 45 neutered).

#### Breeds

There were 92 (72.4%) domestic European cats and 12 (9.4%) Maine Coon cats. Other feline breeds included Persian (6 cases); Ragdoll (3); Carthusian; Exotic and Russian Blue (2); Burmese, Oriental, Persian mix, Sacred of Burma, Scottish straight, Siamese, Siberian and Turkish angora (1) (Table 2).

**Table 2:** **Feline breeds of cases included in the study**.

Breeds	Number of animals (%)
Domestic European	92 (72.4)
Maine Coon	12 (9.4)
Persian	6 (4.7)
Ragdoll	3 (2.3)
Carthusian, Exotic, Russian Bleu	2 (1.6)
Burmese, Oriental, Persian Mix, Sacred of Burma, Scottish Straight, Siamese, Siberian and Turkish Angora	1 (0.8)

#### Body Weight

Mean body weight of the cats was 4.6 ± 1.4 kg (median 4.5, range 2.2–8.5).

#### Vertical Mandibular Range of Motion

The mean vmROM in cats was 62 ± 8 mm (median 63, range 41–84). Minimum (41 mm) and maximum (84 mm) vmROM were recorded in two domestic European cats. Their body weights were 3.1 and 3.5 kg and their age 13 and 156 months, respectively.

**Figure 7:**
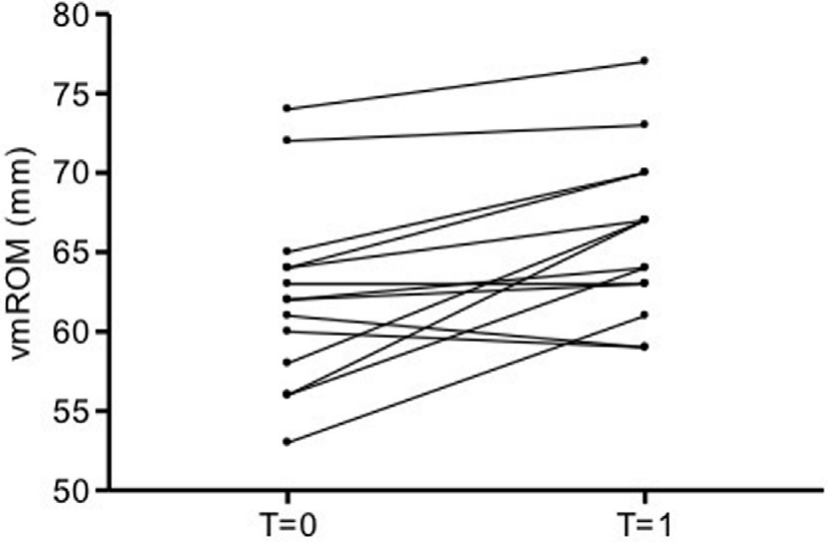
**Before and after plot of vmROM in cats**. vmROM is significantly higher at last admission (*T* = 1) than first admission (*T* = 0) (*p* = 0.003).

Fourteen cats had vmROM measured more than once (mean time interval between first and last visit: 3 months, median: 2 months, range: 1–10 months) (Table S4 in Supplementary Material). The mean vmROM difference between two examination was 4 ± 4 mm (median 3, range −2 to 11). If cats younger than 1 year of age at the time of the first examination (three animals) were excluded, vmROM difference between two examination was 3 ± 4 mm (median 3, range −2 to 11). These three young cats were evaluated between 1 and 10 months apart, with a difference in vmROM between −1 and +9 mm. Differences in vmROM were significant including or not young cats (*p* = 0.003 and *p* = 0.028, respectively; Figure 7). Body weight did not differ between last admission and first admission, in either case.

**Figure 8:**
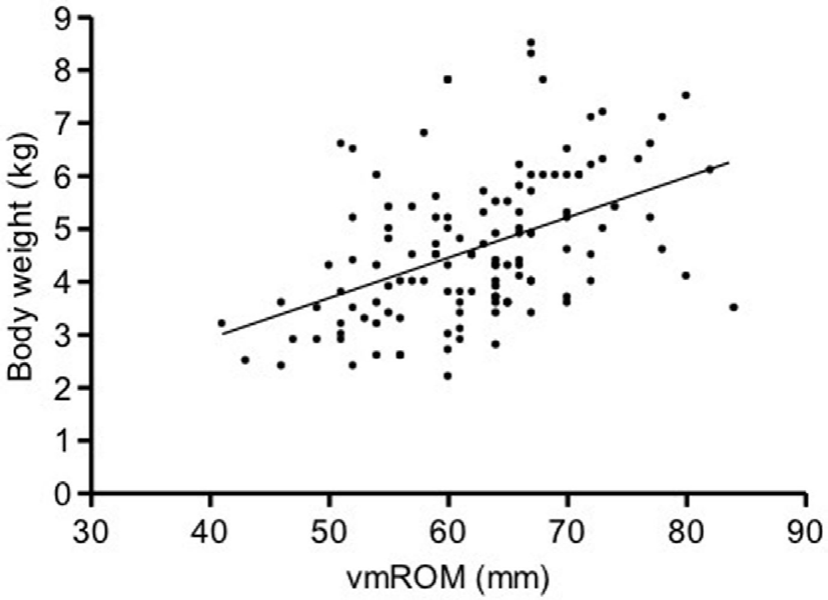
**Scatter dot plot of vmROM and body weight in cats**. A significant positive correlation is identified (*p* < 0.001). The regression line is shown.

**Figure 9:**
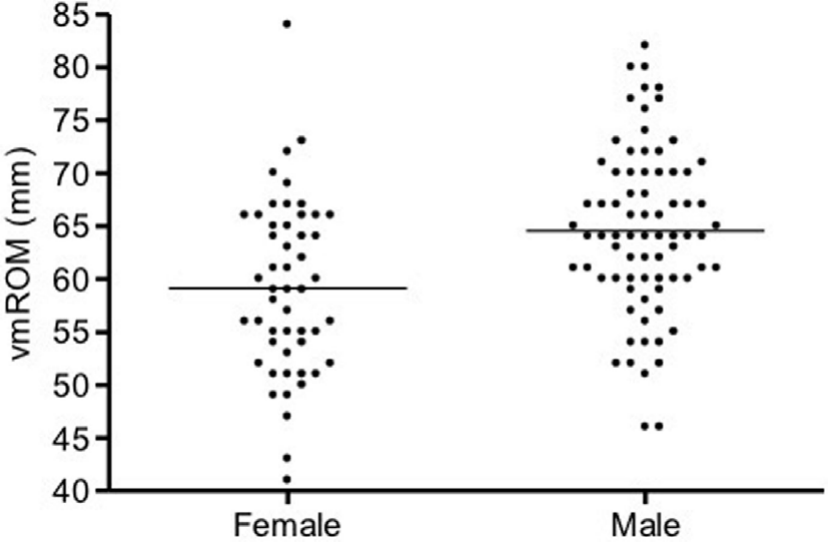
**Scatter dot plot of vmROM in female and male cats**. vmROM is significantly higher in male (*p* < 0.001). The horizontal line depicts the mean.

**Figure 10:**
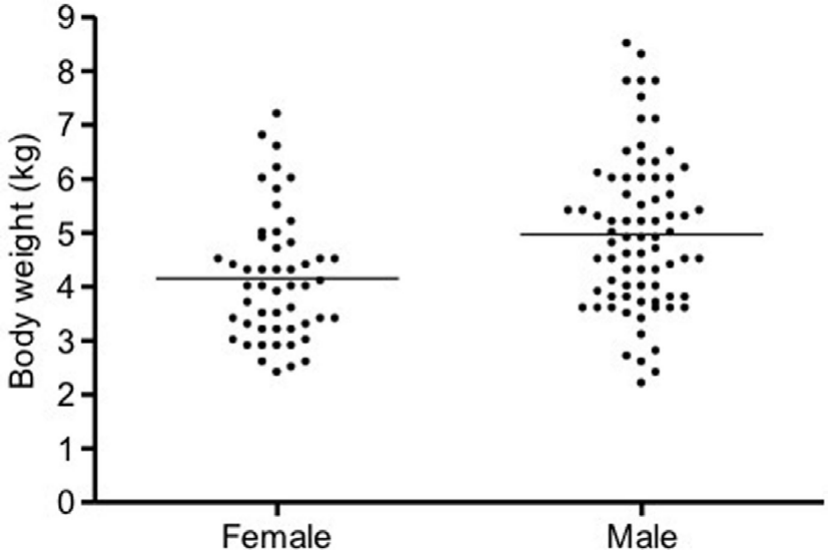
**Scatter dot plot of body weight in female and male cats**. Body weight is significantly heavier in male (*p* < 0.001). The horizontal line depicts the mean.

In cats, a significant positive correlation was documented between vmROM and body weight (*r* = 0.46, CI 95%: 0.31–0.59, *p* < 0.001; Figure 8) and between vmROM and age, although with a low correlation coefficient (*r* = 0.20, CI 95%: 0.02–0.36, *p* = 0.028). vmROM was significantly higher in male than female (male: 65 ± 1 mm, female: 59 ± 1 mm, *p* < 0.001; Figure 9), and body weight of male cats was heavier than in females (male: 5.0 ± 1.4 kg, female: 4.2 ± 1.2 kg, *p* < 0.001; Figure 10).

Vertical mandibular range of motion was not different between domestic European and purebreed cats (*p* = 0.846).

## Discussion

In this study, only the linear measurement of vmROM (i.e., the distance between maxillary and mandibular incisor teeth at maximum mandibular extension) was registered. This value has been shown in humans to be more useful than the measurement of right and left excursion, protrusion, overbite, and overjet, when discriminating between patients with and without TMJ disorders (13). Given the normal anatomy and physiology of the canine and feline joint, allowing limited mandibular movements other than in vertical dimension, vmROM is also likely the most useful clinical measurement in dogs and cats. However, further studies to objectively evaluate the mobility in all dimensions would be necessary to prove it.

Also, only passive ROM was measured, i.e., the mandible was passively moved by the examiners while the patient was under general anesthesia, and the patient’s musculature was relaxed. A difference may exist with active ROM (i.e., the opening of the mouth under voluntary effort, in a vigilant animal). Therefore, the measurements reported here may not be perfectly applicable to patients that are evaluated when awake. However, it has been shown in humans that the difference between active and passive ROM may be so small that its clinical importance may be questioned (14).

Vertical mandibular range of motion measured as described was certainly influenced by the force applied by the assistant to open the mouth, which could not be standardized. In fact, different vmROMs were often recorded for animals visited more than once, at different times. Even if in the great majority of these cases discrepancies were within an acceptable limit of 2 ± 8 mm in dogs and 4 ± 4 mm in cats, sometimes the measurement discrepancy was much higher (up to 29 mm in dogs and 11 mm in cats). In some patients, this difference was likely real and linked to the increasing age and body size. In many other instances, though, these factors were the unlike cause of the registered differences. In fact, even if animals younger than of 1 year of age were eliminated from the statistical evaluation, considering therefore only adult animals, the variation in vmROM was still up to 16 mm in dogs and up to 11 mm in cats. Taking multiple, repeated measurements during the same session could decrease the risk of accidental error and should be considered for future studies and evaluations. Three consecutive measurements are recommended in human patients (15, 16).

The depth of general anesthesia could also have influenced the degree of muscle relaxation and, therefore, the ability to maximally extend the mandible. However, although timing was not specifically standardized, the measurement took place after a deep plane of anesthesia was reached, as indicated by anesthetic parameters. Therefore, the influence of muscle relaxation seems to be an unlikely factor affecting vmROM in this study.

On the other hand, the registered measurements of mandibular extension was likely influenced by the presence/absence of some of the incisor teeth and by the couple of teeth chosen as anatomical landmarks (which was not specified). In some cases, a small discrepancy between two different measurements taken at different times could have been due to the fact that incisors used as landmarks for the first measurement were successively extracted or lost, and the second measurement was possibly taken from a different location.

The overbite (i.e., vertical overlap between maxillary and mandibular incisor teeth) was not considered when registering the maximum vmROM. A small overbite is expected and considered normal both in dogs and cats, with the mandibular incisor teeth occluding on the cingulum of the maxillary counterparts (17, 18). Therefore, the true vmROM was likely slightly higher than reported here. However, when considering the clinical application of our results, the registered measurement was considered to be the value of interest. In other words, rather than evaluating the angular displacement of the mandible relative to the cranium, we were interested in identifying the maximal mouth opening in dogs and cats, as reduction of this space is what causes clinical issues (e.g., inability to eat normally) to the patients.

**Figure 11:**
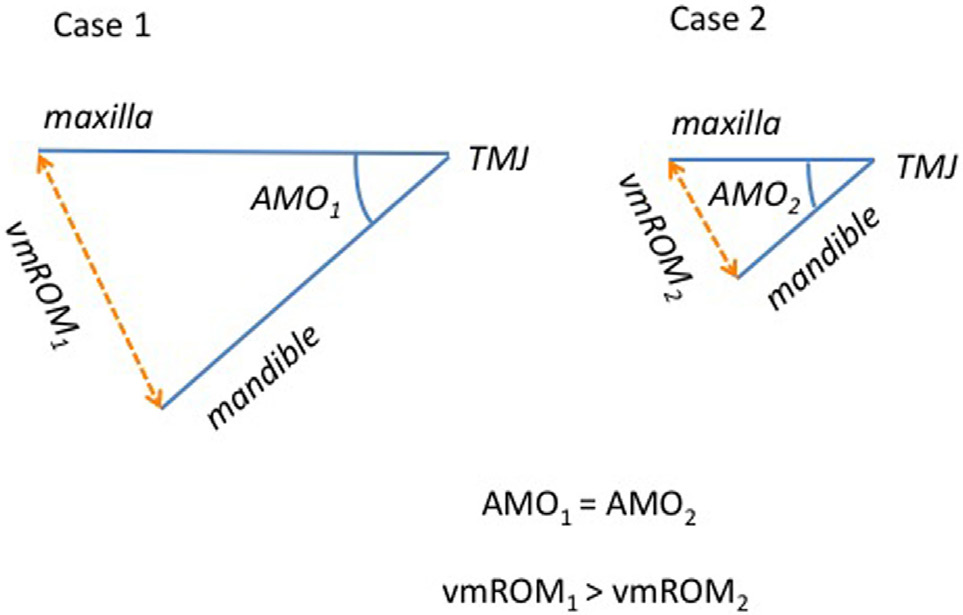
**Schematic representation comparing two individuals with the same angle of mouth opening (AMO) but different mandibular length (and body size)**. The interincisal distance (vmROM) results different, being higher for animals with longer mandibles.

As expected, overall vmROM increased with body weight, and a positive correlation was found between these parameters in cats and in all subgroups of dogs, except subgroup 2. These results can be explained by the differences in mandibular dimensions documented in animals of different body weight and size. In fact, the length of the mandible (i.e., the distance measured from the mandibular condyle to the lower incisor teeth) has been shown to represent an important factor influencing vmROM in humans (19–21). Individuals with the same TMJ mobility and same angle of mouth opening (the angular displacement of the mandible relative to the cranium) may differ considerably with respect to vmROM due to the differences in mandibular length (Figure 11). At the same time, individuals with similar mandibular length may differ significantly in vmROM when the angle of mouth opening differs. Further studies involving cephalometric analysis of the facial skeleton to evaluate mandibular length and angle of mouth opening in dogs and cats of different age, sex, size, and breed (i.e., skull morphology), and influence of these factors into the vertical ROM of the mandible are warranted.

The correlation between vmROM and sex in humans is rather controversial. Men show an average higher vmROM than women, but it has been demonstrated that when the values are adjusted according to body size, the measures become more equal or even higher in women (14, 22, 23).

In the present study, vmROM and body weight were significantly higher in male than female cats. Similarly, overall male dogs were heavier than female dogs and showed a higher vmROM. However, some differences were observed in dog subgroups. A positive correlation could not be demonstrated between body weight and vmROM in subgroup 2, and only in subgroup 4, male dogs were significantly heavier than female. These differences might be explained by the fact that by dividing the studied population in subgroups, the number of cases during each analysis is reduced, easily leading to a type II statistical error. Also, the weight ranges of the subgroups were chosen arbitrarily, and a different choice could have influenced our results. This selection bias could also have had a role in explaining the wide range of vmROM measurements recorded within any single canine subgroup. We still consider our data to be useful and serve as a baseline for future studies and measurements.

It has been shown that, possibly due to physiologic joint (ligamentous) laxity in children, vmROM increases until 10 year of age and then reaches adult level (15, 24). The laxity of the articular capsule and masticatory muscles is supposed to allow for an increased vmROM at a younger age. In the present study, there was no correlation between vmROM and age overall or in any of the canine subgroups. However, the results might be biased by the fact that only a small number of young animals (31 dogs) were evaluated. Also, only seven of the young dogs had measurements taken more than once, registered between 1 and 8 months after the first visit, which may be too short of a period of time to show any significant difference. Interestingly, in the feline species, vmROM increased with age, with the maximum measurement registered in a 13-year-old cat. However, the correlation coefficient was very low. We arbitrarily chose the age of 12 months as the cutoff between young and adult dogs and cats, but further investigation should be carried out, including a larger number of growing animals and involving a number of successive measurements taken at specific ages, to prove that the selected value is the correct threshold, to evaluate if physiologic joint laxity plays a role and varies up until this age or there are breed and species-associated variations.

The lack of diagnostic imaging procedures to confirm the absence of TMJ anomalies and other diseases able to affect vmROM is certainly an important limitation to this study. In fact, the possibility that some animals had an abnormal ROM can not be completely excluded, even if cases with a history of maxillofacial trauma and other diseases that could have affected vmROM were excluded from the study population. The high number of animals included in the study should limit any statistical influence by false negative cases. Nonetheless, further studies evaluating the correlation between the tomographic appearance of the articular and extrarticular structures and vmROM are certainly warranted.

In summary, vmROM increases with body weight, and vmROM is higher in male, either in dogs or cats; vmROM did not correlate with age in dogs and positively correlated, albeit weakly, in cats. Baseline measurements of vmROM are expected to be clinically useful in both species and, thus, the authors recommend vmROM evaluation to be incorporated into every diagnostic examination, as it may be valuable in showing changes over time on any single patient.

## Author Contributions

MG: conception and design of the work; acquisition and interpretation of data for the work; drafting of the work; final approval of the version to be published; and agreement to be accountable for all aspects of the work. EZ: analysis and interpretation of data for the work; drafting of the work and revising it critically for important intellectual content; final approval of the version to be published; and agreement to be accountable for all aspects of the work.

## Conflict of Interest Statement

The authors declare that the research was conducted in the absence of any commercial or financial relationships that could be construed as a potential conflict of interest.

## Footnotes

^1^GraphPad Prism version 4.0, GraphPad Software Inc., La Jolla, CA, USA.
