# Supplementary material for: Vertical Mandibular Range of Motion in Anesthetized Dogs and Cats
Source: Front Vet Sci. 2016 Jun 28;3:51. doi: 10.3389/fvets.2016.00051 (PMC4923261; doi:10.3389/fvets.2016.00051)
Supplement: Supplementary file 1 [file Table_1.PDF]

Supplementary Material

VERTICAL MANDIBULAR RANGE OF MOTION IN ANESTHETIZED DOGS AND CATS

Margherita Gracis[1,2*], Eric Zini[1,3,4]

[1]Istituto Veterinario di Novara, Granozzo con Monticello (Novara), Italy; [2]Clinica Veterinaria San Siro, Milano, Italy; [3]Clinic for Small Animal Internal Medicine, Vetsuisse Faculty, University of Zurich, Zurich, Switzerland; [4]Department of Animal Medicine, Production and Health, University of Padova, Legnaro (Padova), Italy.

*** Correspondence:**
Margherita Gracis

info@margheritagracis.it

Supplementary Table 1. vmROM dogs, ordered by body weight. Re-examinations have been excluded from this table, and the maximum vmROM measurement for each case is shown. Body weight in Kilograms. Sex: F, female; FS female spayed; M, male; MC, male castrated. Age in months. vmROM in millimeters.

Patient ID	Breed	Body weight	Sex	Age	vmROM
1	Chihuahua	1.4	F	14	40
2	Mixed breed	1.4	M	15	43
3	Chihuahua	1.4	FS	8	44
4	Chihuahua	1.8	FS	78	53
5	Chihuahua	1.9	F	8	42
6	Maltese	1.9	MC	102	65
7	Yorkshire Terrier	2.1	F	139	52
8	Chihuahua	2.1	F	8	50
9	Chihuahua	2.2	MC	13	54
10	Shi-Tzu	2.3	F	4	55
11	Chihuahua	2.4	FS	150	48
12	Maltese	2.4	F	12	73
13	Spitz	2.5	M	8	57
14	Spitz	2.6	F	165	56
15	Chihuahua	2.6	M	9	56
16	Yorkshire Terrier	2.6	M	55	70
17	Pugs	2.6	M	4	51

Patient ID	Breed	Body weight	Sex	Age	vmROM
18	Chihuahua	2.7	FS	38	66
19	Chihuahua	2.7	M	113	52
20	Miniature Pinscher	2.7	F	12	62
21	Epagneul Papillon	2.7	F	20	67
22	Yorkshire Terrier	2.8	F	20	73
23	Miniature Pinscher	2.8	M	83	68
24	Maltese	2.8	M	31	78
25	Chihuahua	2.8	M	113	58
26	Mixed breed	2.9	F	71	69
27	Maltese	3.0	M	42	63
28	Chihuahua	3.0	M	8	52
29	Yorkshire Terrier	3.0	M	62	77
30	Miniature Pinscher	3.0	F	6	64
31	Chihuahua	3.0	M	31	67
32	Miniature Poodle	3.1	M	22	82
33	Pekingese dog	3.4	F	119	70
34	Shi-Tzu	3.5	FS	46	71
35	Dachshund	3.6	M	2	83
36	Maltese	3.6	M	37	85
37	Maltese	3.6	MC	65	76
38	Maltese	3.8	FS	107	82

Patient ID	Breed	Body weight	Sex	Age	vmROM
39	Yorkshire Terrier	3.8	M	106	55
40	Jack Russell Terrier	3.8	M	105	100
41	Miniature Pinscher	4.1	M	167	59
42	Maltese	4.2	M	9	79
43	Maltese	4.4	M	86	88
44	Miniature Poodle	4.6	FS	13	95
45	Miniature Poodle	4.8	M	150	79
46	Maltese	4.8	F	73	77
47	Miniature Poodle	4.9	FS	123	84
48	Chihuahua	4.9	M	38	74
49	Yorkshire Terrier	5.0	M	78	92
50	Toy Poodle	5.0	MC	172	86
51	Jack Russell Terrier	5.0	FS	120	93
52	Miniature Poodle	5.1	M	141	82
53	Dachshund	5.3	M	137	76
54	Miniature Pinscher	5.3	FS	124	77
55	Maltese	5.4	M	19	66
56	Fox Terrier	5.4	FS	7	114
57	Yorkshire Terrier	5.4	FS	124	80
58	Miniature Poodle	5.5	FS	158	86

Patient ID	Breed	Body weight	Sex	Age	vmROM
59	Dachshund	5.6	FS	167	89
60	Mixed breed	5.6	M	211	87
61	Dachshund	5.6	FS	71	88
62	Mixed breed	6.0	MC	196	97
63	Mixed breed	6.0	FS	61	94
64	Mixed breed	6.0	M	137	89
65	Mixed breed	6.0	MC	16	98
66	Mixed breed	6.0	FS	182	96
67	Dachshund	6.1	F	77	85
68	Lowchen (Little Lion dog)	6.2	M	10	103
69	Shi-Tzu	6.2	FS	21	74
70	Jack Russell Terrier	6.3	F	27	93
71	Miniature Poodle	6.4	M	60	91
72	Miniature Poodle	6.5	FS	210	101
73	Dachshund	6.5	FS	115	92
74	Shi-Tzu	6.6	M	12	86
75	Shi-Tzu	6.8	M	67	53
76	Mixed breed	7.0	MC	130	98
77	Jack Russell Terrier	7.0	FS	84	87
78	Dachshund	7.2	F	11	99

Patient ID	Breed	Body weight	Sex	Age	vmROM
79	West Highland Terrier	7.2	M	137	110
80	Dachshund	7.3	FS	103	98
81	Mixed breed	7.5	M	110	110
82	Mixed breed	7.5	M	200	85
83	Dachshund	7.7	FS	151	87
84	Mixed breed	7.7	FS	175	109
85	Yorkshire Terrier	7.8	M	95	96
86	Mixed breed	7.9	FS	157	103
87	Jack Russell Terrier	7.9	FS	95	88
88	Dachshund	7.9	FS	179	118
89	Mixed breed	8.2	M	131	104
90	Dachshund	8.3	FS	115	80
91	Mixed breed	8.5	FS	96	93
92	Zwergschanuzer	8.6	M	115	122
93	Jack Russell Terrier	8.6	MC	75	83
94	Miniature Poodle	8.7	M	182	108
95	Cavalier King Charles	9.0	M	36	71
96	Cavalier King Charles	9.2	F	56	74
97	Mixed breed	9.2	MC	44	103

Patient ID	Breed	Body weight	Sex	Age	vmROM
98	Mixed breed	9.3	M	84	112
99	West Highland Terrier	9.3	FS	112	96
100	Mixed breed	9.4	M	166	96
101	American Cocker	9.5	M	36	104
102	Mixed breed	9.6	FS	141	117
103	Mixed breed	9.8	M	115	72
104	Jack Russell Terrier	9.8	M	16	112
105	English Bulldog	10.0	F	4	70
106	French Bouledogue	10.0	M	76	76
107	Mixed breed	10.0	M	134	128
108	Mixed breed	10.2	MC	122	154
109	Fox Terrier	10.4	FS	142	127
110	Bull Terrier	10.5	F	15	78
111	Mixed breed	10.5	MC	130	91
112	Mixed breed	10.5	FS	198	98
113	Dachshund	10.8	FS	131	101
114	Fox terrier	11.0	M	77	127
115	Pugs	11.0	M	128	68
116	Cocker Spaniel	11.1	FS	82	99
117	Cocker Spaniel	11.5	FS	86	118

Patient ID	Breed	Body weight	Sex	Age	vmROM
118	Mixed breed	11.5	M	89	118
119	Mixed breed	11.5	M	126	119
120	Cocker Spaniel	11.5	F	20	108
121	Mixed breed	11.6	FS	82	107
122	Cocker Spaniel	11.8	FS	17	96
123	Cocker Spaniel	12.0	F	87	102
124	Cocker Spaniel	12.2	M	106	112
125	Mixed breed	12.5	FS	31	90
126	Mixed breed	12.9	FS	114	90
127	Mixed breed	13.0	FS	132	104
128	Mixed breed	13.0	M	20	132
129	Beagle	13.5	FS	164	109
130	Mixed breed	13.7	FS	122	99
131	Mixed breed	14.0	FS	172	101
132	Rhodesian Ridgeback	14.0	M	4	112
133	English Setter	14.0	FS	59	74
134	French Bouledogue	14.2	FS	74	59
135	Border Collie	14.6	M	8	135
136	Mixed breed	15.0	M	91	110
137	English Setter	15.0	M	4	116

Patient ID	Breed	Body weight	Sex	Age	vmROM
138	Cocker Spaniel	15.4	M	63	98
139	Tibetan Terrier dog	15.5	FS	46	109
140	Cocker Spaniel	15.5	M	58	118
141	Beagle	15.5	M	61	107
142	American Cocker	16.0	F	94	115
143	Basset Bleu de Guascogne	16.0	M	94	139
144	Mixed breed	16.0	F	146	118
145	Beagle	16.3	M	33	131
146	English Setter	18.0	FS	119	115
147	Mixed breed	18.0	FS	15	115
148	Bull Terrier	18.0	FS	55	98
149	Lagotto	18.0	FS	96	115
150	Mixed breed	18.5	M	21	123
151	Australian shepherd	18.6	F	61	140
152	Belgian Shepherd	19.0	M	47	130
153	Mixed breed	20.0	FS	135	132
154	Mixed breed	21.0	FS	193	138
155	Rottweiler	21.0	M	6	109
156	Cocker Spaniel	21.0	M	75	121
157	Irish Setter	21.0	FS	133	135

Patient ID	Breed	Body weight	Sex	Age	vmROM
158	Labrador Retriever	21.8	F	7	128
159	Border Collie	22.0	FS	60	147
160	Border Collie	22.3	M	24	131
161	Mixed breed	22.5	MC	168	138
162	Bull Terrier	23.0	M	40	91
163	Golden Retriever	23.0	F	7	125
164	Border Collie	23.3	M	30	122
165	Mixed breed	24.0	M	146	128
166	Mixed breed	24.7	FS	15	140
167	American Staffordshire	24.8	MC	41	122
168	Golden Retriever	25.0	FS	33	128
169	Mixed breed	25.0	FS	96	129
170	Mixed breed	25.0	M	145	125
171	Golden Retriever	25.0	M	8	115
172	Mixed breed	25.0	M	49	141
173	Azawakh	25.0	FS	49	105
174	Akita Inu	25.5	M	13	154
175	Mixed breed	25.6	FS	150	118
176	English Setter	26.0	MC	99	113
177	Mixed breed	26.0	MC	30	154

Patient ID	Breed	Body weight	Sex	Age	vmROM
178	Labrador Retriever	26.0	FS	71	129
179	Australian Shepherd	26.0	M	151	131
180	Bernese Mountain dog	26.0	F	5	130
181	Labrador Retriever	26.1	FS	127	112
182	Belgian Malinoise	26.5	F	52	120
183	Mixed breed	26.7	M	109	142
184	Pitbull	27.0	F	69	132
185	Czechoslovakian Wolfdog	27.5	M	60	155
186	Greyhound	27.8	MC	84	134
187	Labrador Retriever	28.0	M	11	111
188	Irish Setter	28.0	FS	137	129
189	Weinmaraner	28.0	F	10	127
190	Boxer	28.0	FS	79	93
191	Mixed breed	28.0	M	81	155
192	Mixed breed	28.0	FS	118	132
193	Mixed breed	28.5	FS	144	132
194	Flatcoated Retriever	28.5	F	17	137
195	Boxer	28.5	FS	92	104
196	Argentine Dogo	28.8	F	15	140

Patient ID	Breed	Body weight	Sex	Age	vmROM
197	Boxer	29.0	FS	90	109
198	Boxer	29.3	F	109	104
199	Australian Shepherd	29.5	MC	31	140
200	German Shepherd	30.0	M	7	141
201	German Shepherd	30.0	FS	103	130
202	Labrador Retriever	30.0	M	7	128
203	Maremma sheepdog	30.0	F	22	160
204	Labrador Retriever	30.0	M	13	134
205	Boxer	30.0	M	43	102
206	American Staffordshire	30.0	FS	118	112
207	Golden Retriever	30.0	FS	31	130
208	Labrador Retriever	30.5	M	83	132
209	Greyhound	31.0	MC	98	120
210	Boxer	31.0	FS	99	100
211	Doberman Pinscher	31.0	F	10	161
212	Boxer	31.0	M	36	95
213	Labrador Retriever	31.0	M	143	131
214	Labrador Retriever	31.0	M	110	125
215	American Staffordshire	31.5	MC	133	150

Patient ID	Breed	Body weight	Sex	Age	vmROM
216	Golden Retriever	32.0	MC	26	151
217	Boxer	32.0	FS	110	106
218	Mixed breed	32.4	M	92	139
219	Boxer	32.5	FS	127	98
220	Labrador Retriever	32.5	FS	94	122
221	Czechoslovakian Wolfdog	33.0	M	25	164
222	Bernese Mountain dog	33.0	F	49	138
223	German Shepherd	33.0	FS	98	131
224	Labrador Retriever	33.3	MC	96	128
225	Golden Retriever	34.0	M	62	150
226	Rhodesian Ridgeback	34.0	F	47	135
227	Golden Retriever	34.0	M	145	117
228	Boxer	34.0	MC	18	124
229	Czechoslovakian Wolfdog	35.0	F	37	142
230	Labrador Retriever	35.0	MC	155	134
231	Mixed breed	35.0	FS	133	148
232	Czechoslovakian Wolfdog	35.0	M	29	155
233	Labrador Retriever	35.0	M	35	136

Patient ID	Breed	Body weight	Sex	Age	vmROM
234	Pitbull	35.0	M	69	146
235	Bernese Mountain dog	35.0	MC	35	160
236	Czechoslovakian Wolfdog	35.0	M	27	155
237	German Shepherd	36.0	F	85	138
238	Rhodesian Ridgeback	37.0	M	134	152
239	Golden Retriever	37.0	F	102	137
240	Labrador Retriever	37.0	M	24	149
241	Rottweiler	37.0	M	17	155
242	Doberman Pinscher	37.0	F	42	125
243	Labrador Retriever	37.0	MC	34	138
244	German Shepherd	37.5	M	15	138
245	Boxer	38.0	M	85	109
246	Golden Retriever	38.0	M	49	124
247	Labrador Retriever	38.0	M	116	156
248	Alaskan Malamute	39.0	M	63	138
249	Labrador Retriever	40.0	M	79	147
250	Great Pyrenees dog	40.0	M	7	155
251	Czechoslovakian Wolfdog	40.0	M	17	152

Patient ID	Breed	Body weight	Sex	Age	vmROM
252	Golden Retriever	40.0	M	138	142
253	Doberman Pinscher	40.0	M	24	165
254	Rottweiler	41.0	M	151	175
255	Alaskan Malamute	44.5	M	33	143
256	Argentine Dogo	45.0	M	47	124
257	Labrador Retriever	46.0	M	112	135
258	Bobtail	48.0	MC	54	93
259	Rottweiler	56.0	M	25	139
260	Irish Wolfhound	69.0	M	69	180
