# Supplementary material for: Vertical Mandibular Range of Motion in Anesthetized Dogs and Cats
Source: Front Vet Sci. 2016 Jun 28;3:51. doi: 10.3389/fvets.2016.00051 (PMC4923261; doi:10.3389/fvets.2016.00051)
Supplement: Supplementary file 2 [file Table_2.PDF]

Supplementary Material

VERTICAL MANDIBULAR RANGE OF MOTION IN ANESTHETIZED DOGS AND CATS

Margherita Gracis[1,2*], Eric Zini[1,3,4]

[1]Istituto Veterinario di Novara, Granozzo con Monticello (Novara), Italy; [2]Clinica Veterinaria San Siro, Milano, Italy; [3]Clinic for Small Animal Internal Medicine, Vetsuisse Faculty, University of Zurich, Zurich, Switzerland; [4]Department of Animal Medicine, Production and Health, University of Padova, Legnaro (Padova), Italy.

*** Correspondence:**
Margherita Gracis

info@margheritagracis.it

Supplementary Table 2. Dogs that had multiple vmROM measured at different times, ordered by the body weight recorded at the time of the first visit. Patient ID: corresponding to patient ID in Supplementary Table 1. Body weight in Kilograms. Sex: F, female; FS female spayed; M, male; MC, male castrated. Age in months. vmROM in millimeters. Highlighted in light green: re-examinations.

Number	Patient ID	Breed	Body weight	Sex	Age	vmROM
1	30	Miniature Pinscher	1.8	F	3	53
	30	Miniature Pinscher	3.0	F	5	62
	30	Miniature Pinscher	3.0	F	6	64
2	11	Chihuahua	2.0	FS	130	43
	11	Chihuahua	2.4	FS	150	48
3	19	Chihuahua	2.4	M	95	50
	19	Chihuahua	2.7	M	113	52
4	20	Miniature Pinscher	2.7	F	12	62
	20	Miniature Pinscher	2.5	F	13	59
	20	Miniature Pinscher	2.7	F	15	62
	20	Miniature Pinscher	2.7	F	16	65
	20	Miniature Pinscher	2.7	F	18	59
	20	Miniature Pinscher	2.8	F	19	59
	20	Miniature Pinscher	2.7	F	20	59
5	24	Maltese	2.8	M	31	78
	24	Maltese	2.8	M	32	78

Number	Patient ID	Breed	Body weight	Sex	Age	vmROM
6	25	Chihuahua	2.8	M	94	57
	25	Chihuahua	2.8	M	113	58
7	36	Maltese	3.5	M	35	79
	36	Maltese	3.6	M	37	85
8	52	Miniature Poodle	5.1	M	141	82
	52	Miniature Poodle	5.7	M	165	76
9	67	Dachshund	6.1	F	77	85
	67	Dachshund	6.2	F	79	78
10	69	Shi-Tzu	6.2	FS	21	74
	69	Shi-Tzu	6.2	FS	22	69
11	68	Lowchen (Little Lion dog)	6.4	M	7	101
	68	Lowchen (Little Lion dog)	6.2	M	10	103
12	71	Miniature Poodle	6.4	M	60	91
	71	Miniature Poodle	6.0	M	77	91
13	73	Dachshund	6.5	FS	115	92
	73	Dachshund	7.0	FS	127	90
14	158	Labrador Retriever	6.7	F	3	99
	158	Labrador Retriever	21.8	F	7	128
15	84	Mixed breed	7.7	FS	175	109
	84	Mixed breed	7.7	FS	176	104

Number	Patient ID	Breed	Body weight	Sex	Age	vmROM
16	89	Mixed breed	8.0	M	129	100
	89	Mixed breed	8.2	M	131	104
17	105	English Bulldog	10,0	F	4	70
	105	English Bulldog	13,6	F	6	69
	105	English Bulldog	16,8	F	8	64
18	107	Mixed breed	10.0	M	131	116
	107	Mixed breed	10.0	M	134	128
	107	Mixed breed	10.8	M	149	124
19	110	Bull Terrier	10.5	F	15	78
	110	Bull Terrier	11.5	F	17	77
20	122	Cocker Spaniel	11.3	FS	15	93
	122	Cocker Spaniel	11.8	FS	17	96
21	200	German Shepherd	12.3	M	3	115
	200	German Shepherd	30.0	M	7	141
22	117	Cocker Spaniel	13.0	FS	48	114
	117	Cocker Spaniel	11.5	FS	86	118
23	130	Mixed breed	13.7	FS	122	99
	130	Mixed breed	13.7	FS	124	90
24	151	Australian shepherd	18.6	F	61	140
	151	Australian shepherd	19.5	F	66	137

Number	Patient ID	Breed	Body weight	Sex	Age	vmROM
25	165	Mixed breed	23.0	M	121	126
	165	Mixed breed	24.0	M	146	128
26	196	Argentine Dogo	24.0	F	14	134
	196	Argentine Dogo	28.8	F	15	140
27	172	Mixed breed	25.0	M	49	141
	172	Mixed breed	25.0	M	70	140
28	176	English Setter	25.4	MC	88	107
	176	English Setter	26.0	MC	99	113
29	174	Akita Inu	25.5	M	12	150
	174	Akita Inu	25.5	M	13	154
30	205	Boxer	26.0	M	40	98
	205	Boxer	30.0	M	43	102
31	183	Mixed breed	26.7	M	109	142
	183	Mixed breed	26.3	M	110	142
32	199	Australian shepherd	27.2	MC	23	129
	199	Australian shepherd	29.5	MC	31	140
33	207	Golden Retriever	30.0	FS	31	130
	207	Golden Retriever	29.0	FS	32	130
34	203	Maremma sheepdog	32.0	F	18	144

Number	Patient ID	Breed	Body weight	Sex	Age	vmROM
	203	Maremma sheepdog	32.0	F	20	160
	203	Maremma sheepdog	30.0	F	22	160
	203	Maremma sheepdog	32.0	F	24	155
35	216	Golden Retriever	32.0	MC	25	145
	216	Golden Retriever	32.0	MC	26	151
36	217	Boxer	32.0	FS	110	106
	217	Boxer	34.0	FS	112	105
37	214	Labrador Retriever	33.0	M	86	122
	214	Labrador Retriever	31.0	M	110	125
38	229	Czechoslovakian Wolfdog	35.0	F	37	142
	229	Czechoslovakian Wolfdog	35.0	F	39	142
39	234	Pitbull	35.0	M	69	146
	234	Pitbull	36.5	M	70	146
40	238	Rhodesian Ridgeback	37.5	M	103	148
	238	Rhodesian Ridgeback	38.1	M	104	150
	238	Rhodesian Ridgeback	37.0	M	134	152

Number	Patient ID	Breed	Body weight	Sex	Age	vmROM
41	245	Boxer	38.0	M	85	109
	245	Boxer	34.0	M	86	101
42	197	Boxer	38.0	FS	78	107
	197	Boxer	38.0	FS	90	109
43	251	Czechoslovakian Wolfdog	40.0	M	17	152
	251	Czechoslovakian Wolfdog	40.0	M	18	145
44	249	Labrador Retriever	40.0	M	79	147
	249	Labrador Retriever	40.0	M	80	145
45	255	Alaskan Malamute	44.5	M	33	143
	255	Alaskan Malamute	44.5	M	34	143
