# Supplementary material for: Vertical Mandibular Range of Motion in Anesthetized Dogs and Cats
Source: Front Vet Sci. 2016 Jun 28;3:51. doi: 10.3389/fvets.2016.00051 (PMC4923261; doi:10.3389/fvets.2016.00051)
Supplement: Supplementary file 3 [file Table_3.PDF]

Supplementary Material

VERTICAL MANDIBULAR RANGE OF MOTION IN ANESTHETIZED DOGS AND CATS

Margherita Gracis[1,2*], Eric Zini[1,3,4]

[1]Istituto Veterinario di Novara, Granozzo con Monticello (Novara), Italy; [2]Clinica Veterinaria San Siro, Milano, Italy; [3]Clinic for Small Animal Internal Medicine, Vetsuisse Faculty, University of Zurich, Zurich, Switzerland; [4]Department of Animal Medicine, Production and Health, University of Padova, Legnaro (Padova), Italy.

*** Correspondence:**
Margherita Gracis

info@margheritagracis.it

Supplementary Table 3. vmROM cats, ordered by body weight. Re-examinations have been excluded from this table, and the maximum vmROM measurement for each case is shown. Body weight in Kilograms. Sex: F, female; FS female spayed; M, male; MC, male castrated. Age in months. vmROM in millimeters.

Patient ID	Breed	Body weight	Sex	Age	vmROM
1	Domestic European	2.2	M	13	60
2	Domestic European	2.4	M	4	46
3	Domestic European	2.4	FS	48	52
4	Persian cat	2.5	FS	48	43
5	Domestic European	2.5	MC	6	56
6	Domestic European	2.6	FS	57	54
7	Domestic European	2.6	FS	28	56
8	Domestic European	2.7	MC	10	60
9	Domestic European	2.8	MC	78	64
10	Persian mix	2.9	F	6	47
11	Exotic	2.9	F	84	49
12	Domestic European	2.9	FS	78	51
13	Domestic European	2.9	FS	123	61
14	Domestic European	3.0	FS	33	51
15	Domestic European	3.0	F	12	60
16	Oriental	3.1	M	6	61
17	Domestic European	3.2	FS	10	54
18	Domestic European	3.2	FS	13	41
19	Persian	3.2	FS	76	51

Patient ID	Breed	Body weight	Sex	Age	vmROM
20	Russian Bleu	3.3	FS	18	56
21	Domestic European	3.3	FS	125	53
22	Domestic European	3.4	FS	13	55
23	Domestic European	3.4	FS	33	61
24	Domestic European	3.4	FS	147	64
25	Domestic European	3.4	MC	11	67
26	Domestic European	3.5	FS	52	49
27	Sacred of Burma	3.5	MC	16	52
28	Domestic European	3.5	FS	156	84
29	Persian cat	3.6	MC	226	64
30	Carthusian	3.6	M	6	46
31	Exotic	3.6	MC	70	54
32	Domestic European	3.6	MC	20	61
33	Domestic European	3.6	FS	36	65
34	Domestic European	3.6	MC	48	65
35	Domestic European	3.6	MC	157	70
36	Domestic European	3.7	FS	150	70
37	Domestic European	3.7	MC	48	64
38	Domestic European	3.7	MC	30	64
39	Domestic European	3.8	MC	132	51
40	Domestic European	3.8	MC	17	60
41	Russian Bleu	3.8	MC	135	61
42	Domestic European	3.8	MC	116	62

Patient ID	Breed	Body weight	Sex	Age	vmROM
43	Domestic European	3.9	FS	61	55
44	Burmese	3.9	MC	143	64
45	Domestic European	4.0	FS	97	56
46	Domestic European	4.0	FS	228	57
47	Persian cat	4.0	MC	187	58
48	Domestic European	4.0	MC	145	64
49	Domestic European	4.0	FS	75	67
50	Maine Coon	4.0	F	15	67
51	Domestic European	4.0	MC	34	72
52	Domestic European	4.1	FS	65	66
53	Domestic European	4.1	MC	65	80
54	Domestic European	4.3	FS	57	50
55	Domestic European	4.3	MC	96	54
56	Ragdoll	4.3	M	9	60
57	Domestic European	4.3	MC	134	64
58	Siberian	4.3	FS	72	64
59	Domestic European	4.3	FS	114	65
60	Domestic European	4.3	FS	38	66
61	Domestic European	4.4	FS	60	66
62	Persian cat	4.4	FS	164	52
63	Domestic European	4.4	MC	71	64
64	Domestic European	4.5	MC	48	57
65	Domestic European	4.5	FS	160	59

Patient ID	Breed	Body weight	Sex	Age	vmROM
66	Persian cat	4.5	FS	120	59
67	Domestic European	4.5	MC	126	62
68	Domestic European	4.5	MC	84	62
69	Maine Coon	4.5	FS	19	62
70	Domestic European	4.5	MC	92	72
71	Domestic European	4.6	MC	58	70
72	Ragdoll	4.6	MC	54	78
73	Domestic European	4.7	MC	13	59
74	Domestic European	4.7	FS	177	63
75	Domestic European	4.8	FS	10	55
76	Domestic European	4.8	MC	58	61
77	Domestic European	4.9	FS	156	66
78	Domestic European	4.9	MC	46	64
79	Carthusian	4.9	MC	145	67
80	Domestic European	4.9	MC	181	67
81	Domestic European	5.0	FS	60	55
82	Domestic European	5.0	FS	177	60
83	Domestic European	5.0	MC	23	66
84	Turkish Angora	5.0	MC	145	73
85	Domestic European	5.2	MC	48	52
86	Maine Coon	5.2	F	71	59
87	Domestic European	5.2	MC	121	60
88	Maine Coon	5.2	M	20	70

Patient ID	Breed	Body weight	Sex	Age	vmROM
89	Domestic European	5.2	MC	128	77
90	Domestic European	5.3	MC	55	63
91	Domestic European	5.3	MC	47	66
92	Domestic European	5.3	MC	72	70
93	Domestic European	5.4	MC	62	55
94	Domestic European	5.4	MC	69	57
95	Domestic European	5.4	MC	36	74
96	Maine Coon	5.5	F	16	64
97	Domestic European	5.5	MC	33	65
98	Domestic European	5.6	MC	132	59
99	Domestic European	5.7	MC	22	63
100	Domestic European	5.7	MC	182	67
101	Maine Coon	5.8	FS	68	66
102	Domestic European	6.0	MC	31	54
103	Domestic European	6.0	FS	56	67
104	Domestic European	6.0	MC	117	68
105	Maine Coon	6.0	FS	23	69
106	Domestic European	6.0	MC	47	70
107	Maine Coon	6.0	MC	17	71
108	Scottish straigth	6.0	MC	77	71
109	Domestic European	6.1	MC	170	82
110	Domestic European	6.2	MC	102	66
111	Maine Coon	6.2	FS	54	72

Patient ID	Breed	Body weight	Sex	Age	vmROM
112	Maine Coon	6.3	MC	16	73
113	Maine Coon	6.3	MC	9	76
114	Domestic European	6.5	MC	31	52
115	Domestic European	6.5	MC	72	70
116	Domestic European	6.6	FS	130	51
117	Domestic European	6.6	MC	99	77
118	Siamese	6.8	FS	117	58
119	Domestic European	7.1	MC	84	72
120	Maine Coon	7.1	MC	15	78
121	Domestic European	7.2	FS	131	73
122	Domestic European	7.5	MC	156	80
123	Domestic European	7.8	MC	120	60
124	Ragdoll	7.8	MC	114	60
125	Domestic European	7.8	MC	96	68
126	Domestic European	8.3	MC	109	67
127	Domestic European	8.5	MC	80	67
