# Supplementary material for: Vertical Mandibular Range of Motion in Anesthetized Dogs and Cats
Source: Front Vet Sci. 2016 Jun 28;3:51. doi: 10.3389/fvets.2016.00051 (PMC4923261; doi:10.3389/fvets.2016.00051)
Supplement: Supplementary file 4 [file Table_4.PDF]

*Supplementary Material*

# VERTICAL MANDIBULAR RANGE OF MOTION IN ANESTHETIZED DOGS AND CATS

**Margherita Gracis[1,2*], Eric Zini[1,3,4]**

[1]Istituto Veterinario di Novara, Granozzo con Monticello (Novara), Italy; [2]Clinica Veterinaria San Siro, Milano, Italy; [3]Clinic for Small Animal Internal Medicine, Vetsuisse Faculty, University of Zurich, Zurich, Switzerland; [4]Department of Animal Medicine, Production and Health, University of Padova, Legnaro (Padova), Italy.

*** Correspondence:**
Margherita Gracis

info@margheritagracis.it

**Supplementary Table 4.** Cats that had multiple vmROM measured at different times, ordered by the body weight recorded at the time of the first visit. Patient ID: corresponding to patient ID in Supplementary Table 3. Body weight in Kilograms. Sex: F, female; FS female spayed; M, male; MC, male castrated. Age in months. vmROM in millimeters. Highlighted in light green: re-examinations.

| Number | Patient ID | Breed | Body weight | Sex | Age | vmROM |
|---|---|---|---|---|---|---|
| 1 | 32 | Domestic European | 2.7 | MC | 10 | 53 |
| | 32 | Domestic European | 3.6 | MC | 20 | 61 |
| 2 | 15 | Domestic European | 3.0 | F | 12 | 60 |
| | 15 | Domestic European | 3.0 | F | 13 | 59 |
| 3 | 23 | Domestic European | 3.4 | FS | 33 | 61 |
| | 23 | Domestic European | 3.0 | FS | 41 | 59 |
| 4 | 25 | Domestic European | 3.6 | MC | 9 | 58 |
| | 25 | Domestic European | 3.9 | MC | 10 | 63 |
| | 25 | Domestic European | 3.4 | MC | 11 | 67 |
| 5 | 36 | Domestic European | 3.9 | FS | 149 | 64 |
| | 36 | Domestic European | 3.7 | FS | 150 | 70 |
| 6 | 63 | Domestic European | 4.4 | MC | 70 | 62 |
| | 63 | Domestic European | 4.4 | MC | 71 | 64 |
| 7 | 74 | Domestic European | 4.6 | FS | 176 | 62 |
| | 74 | Domestic European | 4.7 | FS | 177 | 63 |
| 8 | 84 | Turkish Angora | 4.7 | MC | 144 | 72 |
| | 84 | Turkish Angora | 5.0 | MC | 145 | 73 |
| 9 | 71 | Domestic European | 4.8 | MC | 57 | 65 |

| Number | Patient ID | Breed | Body weight | Sex | Age | vmROM |
|---|---|---|---|---|---|---|
| | 71 | Domestic European | 4.6 | MC | 58 | 70 |
| 10 | 79 | Carthusian | 4.9 | MC | 142 | 56 |
| | 79 | Carthusian | 5.0 | MC | 144 | 59 |
| | 79 | Carthusian | 4.9 | MC | 145 | 67 |
| 11 | 80 | Domestic European | 5.5 | MC | 179 | 64 |
| | 80 | Domestic European | 4.9 | MC | 181 | 67 |
| 12 | 96 | Maine Coon | 5.5 | F | 13 | 56 |
| | 96 | Maine Coon | 5.5 | F | 16 | 64 |
| 13 | 99 | Domestic European | 5.7 | MC | 22 | 63 |
| | 99 | Domestic European | 6.0 | MC | 24 | 63 |
| 14 | 117 | Domestic European | 7.0 | MC | 96 | 74 |
| | 117 | Domestic European | 6.6 | MC | 99 | 77 |
